# The Gratton effect remains after controlling for contingencies and stimulus repetitions

**DOI:** 10.3389/fpsyg.2014.01207

**Published:** 2014-10-24

**Authors:** Chris Blais, Aikaterini Stefanidi, Gene A. Brewer

**Affiliations:** ^1^Department of Psychology, Arizona State UniversityTempe, AZ, USA; ^2^Department of Communications, Arizona State UniversityTempe, AZ, USA; ^3^Center for Strategic Communication, Arizona State UniversityTempe, AZ, USA

**Keywords:** conflict monitoring, cognitive control, cognitive control mechanisms, Stroop effect, conflict adaptation, Gratton effect, feature binding

## Abstract

**Highlights:**

- The conflict monitoring hypothesis signals the need for cognitive control
- The Gratton effect is a key result attributed to the conflict monitoring hypothesis
- Some argue that controlling binding confounds eliminates the Gratton effect

A Gratton effect remains in a vocal Stroop task after eliminating confounds The Gratton effect, the observation that the size of the Stroop effect is larger following a congruent trial compared to an incongruent trial, is one pivotal observation in support of the conflict-monitoring hypothesis. Previous reports have demonstrated that non-conflict components, such as feature binding, also contribute to this effect. Critically, Schmidt and De Houwer (2011) report a flanker task and a button-press Stroop task suggesting that there is no conflict adaptation in the Gratton effect; it is entirely caused by feature binding. The current investigation attempts to replicate and extend this important finding across two experiments using a canonical four-choice Stroop task with vocal responses. In contrast to Schmidt and De Houwer, we observe reliable conflict adaptation after controlling for feature binding. We argue that the overall strength of conflict is critical for determining whether a conflict adaptation component will remain in the Gratton effect after explaining binding components.

## Introduction

The Gratton effect (Gratton et al., 1992) refers to the finding that congruency effects (i.e., Stroop and flanker effects) are reduced following incongruent trials compared to congruent trials. The most widespread explanation of the Gratton effect is the conflict adaptation hypothesis (Carter et al., 1998; Botvinick et al., 2001, 2004), which states that response conflict from the previous trial signals a *need for control* that manifests as a modulation of response times and error rates on the subsequent trial. The net result is that the size of the Stroop effect is larger following a congruent trial than following an incongruent trial. First observed in a flanker task (Eriksen and Eriksen, 1974), it has been observed in many conflict tasks such as Stroop (Kerns et al., 2004; Mayr and Awh, 2009) and Simon (Akçay and Hazeltine, 2007).

An account of the Gratton effect based solely on conflict adaption is unlikely. Others have pointed out that binding effects, namely feature repetition biases, also contribute to the Gratton effect (Mayr et al., 2003; Hommel et al., 2004; Mayr and Awh, 2009). While there is considerable debate in the field, most would acknowledge that any Gratton effect that remains after all binding sources have been eliminated is consistent with conflict adaptation.

Schmidt and De Houwer (2011) were the first to assess two different binding effects, namely contingency biases and congruency switch costs. They reported a Stroop and a flanker experiment in which they eliminated three possible binding confounds: (1) feature repetition biases, (2) sequential contingency biases, and (3) congruency switch costs. Controlling the first two biases was sufficient to eliminate the Gratton effect in RTs for both tasks, and errors in the flanker task. They attributed the remaining Gratton effect in the error data for the Stroop task to a congruency switch cost by showing that there was no congruency repetition by congruency interaction (see below). Thus, the critical question is whether there is indeed a Gratton effect after such sources have been eliminated.

The implications of this finding are profound. The Gratton effect is one of the key findings in support of the conflict-monitoring hypothesis (Carter et al., 1998), and demonstrating that the Gratton effect is entirely the result of feature binding would necessitate reinterpreting literally hundreds of experiments. Therefore, the current paper reviews the three binding confounds, citing literature which shows that no one confound alone can explain the Gratton effect. We then attempt to replicate Schmidt and De Houwer (2011), first by reanalyzing data from a large-scale Stroop study (Blais et al., 2010), and second with a new experiment. In contrast to the findings reported by Schmidt and De Houwer, a robust Gratton effect is observed reasserting that, at least in some tasks, conflict adaptation does contribute to the size of the Gratton effect.

Schmidt and De Houwer (2011) provided an in depth discussion of the following three confounds. They are briefly outlined here to orient readers to the issues.

### Feature repetitions

Following Mayr et al.'s (2003) seminal paper demonstrating that feature repetitions cause the Gratton effect, it has become standard practice to, at the very least, eliminate complete repetitions when both the target feature and distractor feature repeat on the next trial. To eliminate all possible sources of feature repetitions (i.e., target->target, distractor->distractor, target->distractor, and distractor->target transitions), it is necessary to use at least a four-choice task. Studies that have used both a four-choice task, and eliminated all the feature repetition conditions tend to show that, although the Gratton effect is reduced in size, it is not eliminated (e.g., in a Simon task: Akçay and Hazeltine, 2007; in a flanker task: Verbruggen et al., 2006).

### Contingency confounds

Contingency biases are another confound that may also increase the size of the Gratton effect. Participants are often presented with color words in their congruent color more often than would be expected by chance. These types of contingencies are problematic because participants learn them and end up responding faster to the high contingency trials (i.e., when the word is presented in its most frequent color) compared to the low contingency trials (i.e., when the word is presented in a color other than its more frequent color).

Mayr et al. (2003) manipulated proportion congruency between subjects in a flanker task and reported an increase in the size of the Gratton effect. In addition, Schmidt et al. (2007) had subjects identify the color of a non-color word (e.g., MOVE). Critically, the authors systematically paired how often each word would appear in a specific color to create high and low contingency items. For example, in the 75% contingent condition, MOVE might appear in orange on 75% of trials and in red, blue, or green on the remaining 25% of trials. Although there is no conflict *per se*, in addition to finding contingency effects (i.e., subjects were faster to press the orange key if the word was MOVE compared to any other word), they also observed a pseudo-Gratton effect. That is, the contingency effect was larger if preceded by a high contingency item in comparison to a low contingency item.

### Congruency switch costs

Schmidt and De Houwer (2011) proposed the congruency switch hypothesis, a novel third confound against the conflict adaptation account of the Gratton effect. The logic is similar to the task-switch hypothesis (e.g., Monsell, 2003). In short, for an incongruent trial, the system must select between two activated response codes, and bind one to the color and the other to the word. But, for congruent trials, the system simply binds the one activated response code to both the color and the word. Thus, it is conceivable that the system must be reconfigured to respond to a congruent trial compared to an incongruent trial. If so, there may be a cost associated with it. More generally, if even slightly different strategies are used in response to congruent vs. incongruent trials, then switching from one trial type to the next may incur a cost.

To test if a congruency switch is contributing to the Gratton effect, Schmidt and De Houwer (2011) suggest that “analyzing congruency as a function of switch rather than n-1 congruency should lead to roughly additive effect of congruency and switch.” (p. 179). They noted that visual inspection of Freitas et al. (2007) seems to support this hypothesis.

## Experiment 1: a gratton analyses of Blais et al. (2010)

To summarize, there are at least three binding confounds that preclude a conflict adaption hypothesis of the Gratton effect. The evidence in the literature suggests that (1) feature repetitions alone cannot account for a Gratton effect, (2) contingency effects can lead to a pseudo-Gratton effect, and (3) there is speculative evidence that congruency switch costs can yield a Gratton effect.

The current paper reports a series of new analyses from a recent large-scale Stroop study which looked at the role of one's awareness of the proportion of congruent trials on the size of the Stroop effect (Blais et al., 2010). Two important findings emerge. First, when there is no contingency between the color and the word (the 25% congruent condition), there is still a strong Gratton effect after stimulus repetition trials are removed that cannot be explained by a congruency switch. Second, across the entire range of proportions between the 10 and 80% range, the size of the Gratton effect is statistically equal both when stimulus repetition trials are included, and excluded, from the analysis. This finding suggests that color-word contingency plays no role in the Gratton effect.

### Method

For full methodological details, see Blais et al. (2010). Briefly, fifteen subjects spent 8–10 h in the lab performing 19,000 trials in a vocal Stroop task. These trials were administered in blocks of 100 trials across 19 different proportion congruency conditions ranging from 5 to 95% in increments of 5. The order of the 190 blocks was randomized, and each participant received the same order. Each subject responded vocally to the color (RED, BLUE, YELLOW, or GREEN) that the word (red, blue, yellow, or green) was presented in. Stimuli for each block were sampled randomly *with* replacement from the set of 16 possible stimuli such that, if the proportion congruency level was 30%, then 30 congruent stimuli were selected, followed by 70 incongruent stimuli. These 100 items were then randomly sorted and presented to the subject.

It is important to note that 9 of these subjects were asked to estimate the proportion of congruent trials and rate their confidence of this estimate following each block of 100 trials. There was no difference between these two groups on any of the analysis reported here, and so they are treated as a homogenous group of 15 subjects. Since this is a four-choice task, there is no contingency between the color and the word (i.e., the word green is equally likely to appear in any of the four colors) in the 25% congruent condition.

The same correct RTs data as in the original report were used. That is, correct RTs longer than 2000 ms (outliers) or shorter than 200 ms (anticipatory) were excluded, along with any RTs more than 2.5 standard deviations away from the mean within each subject by block by congruency cell.

## Results

### Analyses of RTs

#### Including stimulus repetitions

Table 1 shows the mean RT for the four previous congruency-by-congruency cells at each level of proportion congruency. To maximize power, a 2 (previous congruency) by 2 (current congruency) ANOVA was conducted separately for each proportion condition. The results of this analysis are on the bottom portion of Table 1. To summarize, there was a significant main effect of congruency at each level of proportion congruency. There was a significant main effect of previous congruency at all levels of proportion except [15, 70, 75]. Critically, these factors interacted to produce a Gratton effect at all levels of proportion except [10].

**Table 1:** **Response times for each of the four 2 (previous congruency) × 2 (congruency) cells as a function of the proportion of congruent trials**.

**Condition**		**Proportion of congruent trials (%)**														
		**10**	**15**	**20**	**25**	**30**	**35**	**40**	**45**	**50**	**55**	**60**	**65**	**70**	**75**	**80**
Congruent-congruent		610	608	633	640	614	622	609	621	611	601	592	592	592	594	579
Congruent—incongruent		692	701	723	727	714	712	715	739	727	720	709	721	724	740	730
Incongruent-congruent		644	641	668	671	651	646	636	650	642	631	621	627	622	624	616
Incongruent-incongruent		700	693	718	724	715	706	708	727	714	711	706	710	708	729	726
**ANOVA EFFECTS**																
Previous congruency	*F*_(1, 14)_	**4.69**	3.38	**17.97**	**5.89**	**20.08**	**5.05**	**9.15**	**7.33**	**5.65**	**10.22**	**8.37**	**5.12**	2.10	2.20	**5.14**
	*p*	**0.048**	0.087	**<0.001**	**0.029**	**<0.001**	**0.041**	**0.009**	**0.017**	**0.032**	**0.006**	**0.012**	**0.040**	0.169	0.160	**0.040**
	mean ± std error	**21 ± 9**	13 ± 7	**15 ± 3**	**13 ± 5**	**19 ± 4**	**9 ± 4**	**10 ± 3**	**8 ± 3**	**9 ± 4**	**10 ± 3**	**13 ± 4**	**12 ± 5**	7 ± 5	10 ± 6	**17 ± 7**
Congruency	*F*_(1, 14)_	**30.95**	**42.15**	**53.97**	**60.97**	**87.12**	**68.23**	**56.16**	**79.16**	**80.32**	**84.12**	**109.24**	**95.80**	**102.22**	**86.27**	**79.13**
	*p*	**<0.001**	**<0.001**	**<0.001**	**<0.001**	**<0.001**	**<0.001**	**<0.001**	**<0.001**	**<0.001**	**<0.001**	**<0.001**	**<0.001**	**<0.001**	**<0.001**	**<0.001**
	mean ± std error	**69 ± 12**	**72 ± 11**	**70 ± 9**	**70 ± 9**	**82 ± 8**	**75 ± 9**	**89 ± 11**	**97 ± 11**	**93 ± 10**	**100 ± 10**	**101 ± 9**	**105 ± 10**	**110 ± 10**	**126 ± 13**	**130 ± 14**
Previous congruency × Congruency (Gratton Effect)	*F*_(1, 14)_	3.11	**11.90**	**18.65**	**13.59**	**14.38**	**21.92**	**13.88**	**32.76**	**51.43**	**20.89**	**19.14**	**19.81**	**34.01**	**18.05**	**9.43**
	*p*	0.100	**0.004**	**<0.001**	**0.002**	**0.002**	**<0.001**	**0.002**	**<0.001**	**<0.001**	**<0.001**	**<0.001**	**<0.001**	**<0.001**	**<0.001**	**0.008**
	mean ± std error	27 ± 15	**41 ± 11**	**40 ± 9**	**34 ± 9**	**36 ± 9**	**31 ± 6**	**34 ± 9**	**40 ± 7**	**44 ± 6**	**39 ± 8**	**32 ± 7**	**46 ± 10**	**46 ± 8**	**40 ± 9**	**41 ± 13**

*The bottom portion of the table lists the parameter estimates obtained from the ANOVA testing for the presence of a Gratton effect*.

*The bold values signify that p < 0.05*.

**Figure 1:**
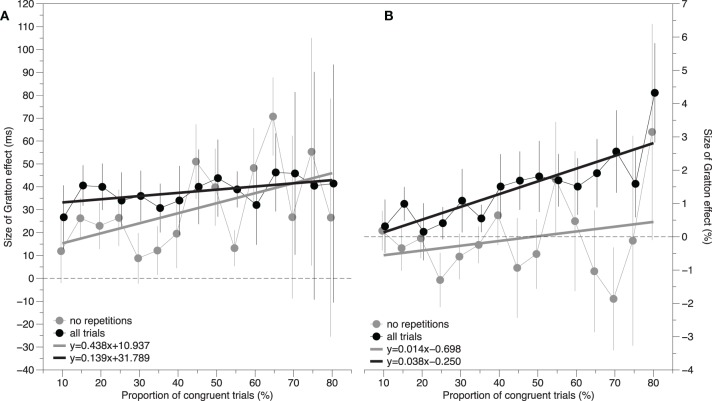
**The size of the Gratton effect as a function of the proportion of congruent trials**. Black lines represent data in which all trials are included in the estimate. Gray lines represent data in which only the trials without feature repetitions are included in the estimate. The panel to the left **(A)** reflects response time difference scores and the panel to the right **(B)** reflects error rate difference scores. A positive slope is consistent with the hypothesis that the contingency between the color and the word contributes to the size of the Gratton effect. Error bars represent the standard error of the mean.

The solid black circles in Figure 1A show the size of the Gratton effect as a function of the proportion of congruent trials. According to pure contingency accounts of the Gratton effect, an increase in the contingency between the color and the word should result in an increase in the size of the Gratton effect. Even though the trend lines appear relatively flat, a repeated measures regression analysis was performed to check for the presence of a non-zero, positive, slope. Specifically, a slope and intercept estimate was calculated for each subject, and a one-sample *t*-test was conducted on these estimates. Conceptually, a positive slope indicates that the Gratton effect increases as the proportion of congruent trials increases. The results of this reveal a slope of 0.139 ± 0.131 ms, *t*_(14)_ = 1.07, *p* > 0.30, and an intercept of 31.789 ± 7.701, *t*_(14)_ = 4.13, *p* < 0.001. This is shown as the solid black line in Figure 1A. Thus, the proportion of congruent trials does not impact the size of the Gratton effect.

#### Excluding stimulus repetitions

Table 2 further divides the mean RT from the four previous congruency x congruency cells in Table 1 into the 15 cells that comprise the non-orthogonal feature repetition types (word->word, color->color, word->color, color->word). In this four-choice task, this amounts to keeping approximately 56% of trials per subject. The same set of ANOVAs reported above were conducted on the cells for which there are no stimulus-repetitions, indicated by the bolded rows in the Table 2. The results are reported in the middle portion of Table 2. There was a significant main effect of congruency at each level of proportion congruency. There was a significant main effect of previous congruency at all levels of proportion except [60, 65, 70, 80]. The Gratton effect was only significant for the [15, 20, 45, 50, 60, 65] conditions.

**Table 2:** **Response times for each of the four 2 (previous congruency) × 2 (congruency) cells as a function of the proportion of congruent trials after dividing them into whether they contain target->target, distractor->distractor, target->distractor, or distractor->target repetitions**.

**Condition**		**Repetition type**				**Proportion of congruent trials (%)**														
		**wW**	**cC**	**wC**	**cW**	**10**	**15**	**20**	**25**	**30**	**35**	**40**	**45**	**50**	**55**	**60**	**65**	**70**	**75**	**80**
**CONGRUENT–CONGRUENT**																				
^(1)^	BLUE_blue_→RED_red_					**629**	**627**	**659**	**666**	**636**	**651**	**634**	**646**	**635**	**624**	**614**	**614**	**613**	**614**	**597**
^(2)^	BLUE_blue_→BLUE_blue_	×	×	×	×	557	531	552	567	549	539	533	553	537	533	527	530	526	532	524
**CONGRUENT–INCONGRUENT**																				
^(3)^	BLUE_blue_→RED_green_					**727**	**741**	**758**	**754**	**750**	**741**	**749**	**764**	**762**	**752**	**743**	**753**	**746**	**772**	**751**
^(4)^	BLUE_blue_→BLUE_red_	×			×	709	715	733	735	717	721	724	736	732	733	713	721	730	736	729
^(5)^	BLUE_blue_→RED_blue_		×	×		613	620	647	669	644	655	644	690	653	657	651	661	671	677	689
**INCONGRUENT–CONGRUENT**																				
^(6)^	RED_blue_→GREEN_green_					**673**	**670**	**701**	**692**	**674**	**665**	**655**	**675**	**670**	**652**	**641**	**653**	**641**	**645**	**638**
^(7)^	RED_blue_→RED_red_	×		×		676	673	690	695	678	687	659	676	664	662	654	658	655	651	642
^(8)^	RED_blue_→BLUE_blue_		×		×	553	554	577	600	580	569	569	571	566	564	549	549	551	556	547
**INCONGRUENT–INCONGRUENT**																				
^(9)^	RED_blue_→GREEN_yellow_					**748**	**737**	**760**	**765**	**753**	**755**	**753**	**766**	**757**	**768**	**739**	**758**	**737**	**770**	**755**
^(10)^	RED_blue_→RED_green_	×				729	720	747	753	742	741	740	764	748	735	745	779	733	764	781
^(11)^	RED_blue_→GREEN_blue_		×			601	600	625	635	611	608	601	639	617	611	610	608	604	642	594
^(12)^	RED_blue_→RED_blue_	×	×			570	556	590	597	590	581	569	586	568	564	567	558	573	580	592
^(13)^	RED_blue_→GREEN_red_			×		749	743	763	769	770	753	752	765	760	760	764	768	787	814	763
^(14)^	RED_blue_→BLUE_green_				×	725	719	738	743	739	728	742	762	762	743	745	718	739	735	738
^(15)^	RED_blue_→BLUE_red_			×	×	726	725	766	769	739	733	748	765	743	769	741	732	755	724	785
**GRATTON ANOVA EFFECTS**																				
Previous congruency		*F*_(1, 14)_				**21.36**	**23.41**	**27.19**	**10.34**	**10.11**	**7.40**	**5.81**	**12.17**	**10.64**	**16.92**	3.29	3.61	0.95	**5.34**	0.12
		*p*				**<0.001**	**<0.001**	**<0.001**	**0.006**	**0.007**	**0.017**	**0.030**	**0.004**	**0.006**	**0.001**	0.091	0.078	0.346	**0.037**	0.731
		mean ± std error				**25 ± 5**	**31 ± 6**	**24 ± 4**	**25 ± 8**	**16 ± 5**	**20 ± 7**	**23 ± 9**	**27 ± 7**	**29 ± 9**	**40 ± 9**	19 ± 10	15 ± 7	18 ± 18	**34 ± 14**	−12 ± 34
Congruency		*F*_(1, 14)_				**56.71**	**62.26**	**76.04**	**49.4**	**60.46**	**68.06**	**57.41**	**60.69**	**66.25**	**47.92**	**64.06**	**58.91**	**76.1**	**50.11**	**30.71**
		*p*				**<0.001**	**<0.001**	**<0.001**	**<0.001**	**<0.001**	**<0.001**	**<0.001**	**<0.001**	**<0.001**	**<0.001**	**<0.001**	**<0.001**	**<0.001**	**<0.001**	**<0.001**
		mean ± std error				**153 ± 20**	**150 ± 18**	**146 ± 16**	**143 ± 20**	**147 ± 18**	**153 ± 18**	**162 ± 21**	**153 ± 19**	**160 ± 19**	**164 ± 23**	**154 ± 19**	**186 ± 23**	**147 ± 16**	**156 ± 21**	**175 ± 31**
Previous congruency × Congruency (Gratton effect)		*F*_(1, 14)_				0.68	**8.26**	**4.77**	4.31	0.59	1.22	1.57	**9.14**	**5.23**	2.62	**7.18**	**16.04**	0.53	1.15	0.24
		*p*				0.424	**0.012**	**0.046**	0.057	0.455	0.288	0.231	**0.009**	**0.038**	0.128	**0.018**	**0.001**	0.479	0.301	0.629
		mean±std error				12 ± 14	**26 ± 9**	**23 ± 10**	26 ± 12	9 ± 11	12 ± 11	20 ± 15	**51 ± 16**	**40 ± 17**	13 ± 8	**48 ± 17**	**71 ± 17**	27 ± 36	55 ± 50	27 ± 52
**CONGRUENCY SWITCH ANOVA EFFECTS**																				
Switch		*F*_(1, 14)_				1.33	**6.07**	**19.04**	0.75	**9.20**	0.01	1.35	**4.68**	**10.88**	1.14	**5.30**	**7.05**	2.99	3.95	1.17
		*p*				0.267	**0.027**	**<0.001**	0.402	**0.009**	0.944	0.265	**0.048**	**0.005**	0.304	**0.037**	**0.019**	0.106	0.067	0.298
		mean ± std error				11 ± 10	**23 ± 9**	**20 ± 5**	7 ± 8	**17 ± 6**	0 ± 4	8 ± 7	**14 ± 6**	**20 ± 6**	6 ± 6	**15 ± 6**	**17 ± 6**	18 ± 11	16 ± 8	18 ± 17
Congruency		*F*_(1, 14)_				**45.95**	**38.46**	**33.92**	**48.58**	**77.61**	**56.43**	**62.05**	**60.48**	**81.94**	**51.83**	**67.71**	**67.99**	**68.45**	**58.02**	**92.03**
		*p*				**<0.001**	**<0.001**	**<0.001**	**<0.001**	**<0.001**	**<0.001**	**<0.001**	**<0.001**	**<0.001**	**<0.001**	**<0.001**	**<0.001**	**<0.001**	**<0.001**	**<0.001**
		mean ±std error				**86 ± 13**	**90 ± 15**	**79 ± 14**	**81 ± 12**	**97 ± 11**	**90 ± 12**	**107 ± 14**	**105 ± 13**	**107 ± 12**	**122 ± 17**	**114 ± 14**	**122 ± 15**	**115 ± 14**	**141 ± 19**	**136 ± 14**
Switch × Congruency (Switch effect)		*F*_(1, 14)_				**6.33**	**5.96**	**7.20**	**5.02**	**15.50**	**5.26**	**5.08**	**5.82**	**8.13**	**19.70**	3.25	**12.68**	1.09	2.45	3.88
		*p*				**0.025**	**0.028**	**0.018**	**0.042**	**0.001**	**0.038**	**0.041**	**0.030**	**0.013**	**<0.001**	0.093	**0.003**	0.313	0.140	0.069
		mean ± std error				**65 ± 26**	**40 ± 16**	**43 ± 16**	**37 ± 17**	**41 ± 10**	**27 ± 12**	**26 ± 12**	**31 ± 13**	**30 ± 10**	**45 ± 10**	23 ± 13	**45 ± 13**	20 ± 19	29 ± 18	45 ± 23

*The middle portion of the table lists the parameter estimates obtained from the ANOVA testing for the presence of a Gratton effect, and the bottom portion of the table lists the parameter estimates obtained from the ANOVA testing whether the remaining Gratton effect results from the congruency switching hypothesis. Note: The absence of an interaction in the bottom portion is consistent with the switching hypothesis. The bold values signify that *p* < *0.05**.

Although many of the Gratton effects were no longer statistically significant, paired *t*-tests showed that the reduction in the size of the Gratton effect from Tables 1, 2 was only reliable for the [55], *p* < 0.027, condition, and was marginal in the [30], *p* = 0.070, condition. For all other comparisons, *p*s > 0.15. For the present purposes, the fate of the 25% congruent condition is most critical, although the size of the Gratton effect was marginal, *p* = 0.057, two-tailed, it was not statistically smaller than when repetitions were included. That is, the 34 ± 9 ms Gratton effect in Table 1 was statistically equivalent to the 26 ± 12 ms Gratton effect in Table 2, *p* > 0.50.

The solid gray circles in Figure 1A show the size of the Gratton effect as a function of the proportion of congruent trials. The same repeated measures regression analysis reported above yielded a slope of 0.438 ± 0.382, *t*_(14)_ = 1.15, *p* > 0.25, and an intercept of 10.937 ± 13.026, *t*_(14)_ = 0.84, *p* > 0.40. This is shown as the dotted gray line in Figure 1A. Although the slope is numerically larger and the intercept is numerically smaller than in the previous analysis that included repetition effects, both are statistically equivalent to the previous parameter estimates (*p* = 0.441 for the slope, and *p* = 0.111 for the intercept).

#### Can the switch hypothesis account for the remaining gratton effect?

Even though the size of the Gratton effects were statistically equivalent across most of the proportion conditions, visual inspection of Figure 1 suggests that, generally, the Gratton effect is numerically smaller when repetitions are removed. To assess whether the remaining Gratton effect is the result of congruency switching (i.e., Schmidt and De Houwer, 2011), a 2 (Switch) × 2 (Congruency) analysis was conducted at each proportion level. The results of this analysis are shown on the bottom proportion of Table 2. An in-depth description of the logic of this analysis is provided by Schmidt and De Houwer (2011). Briefly, additive effects of congruency switch (whether the congruency on the previous trial is the same, or different, on the current trials) and congruency are consistent with an interpretation in which the Gratton effect results from a reconfiguration switch cost. An interaction of these factors cannot rule out a conflict adaption account of the Gratton effect. There was a main effect of congruency at all levels of proportion. There was a switch cost at the following levels of proportion [15, 20, 30, 40, 45, 60, 65]. Critically, these factors interact, rather than add, at each level of proportion except [60, 70, 75, 80]. Furthermore, and even at the levels of proportion at which they fail to interact, the effect seems too large to attribute to purely additive factors.

### Analyses of errors

Despite a very low error rate of 2.1%, for the sake of completeness, the entire set of analyses performed on RTs was also done on error rates. It should be noted that the lack of a main effect of congruency effect in many of the analyses, make it difficult to interpret “higher order” effects, such as the Gratton effect, or the increase in the size of the Gratton effect as the proportion of trials increases. It should also be noted that the same analyses performed on efficiency scores (RT divided by accuracy for each cell; Townsend and Ashby, 1983) yields effects which completely replicate the analyses on the RT data.

#### Including stimulus repetitions

Table 3 mirrors Table 1 with mean percent error rate in place of RTs. The same analysis contained in Table 1 is shown at the bottom of Table 3. There was a significant main effect of congruency at each level of proportion congruency except [30]. There was only a significant main effect of previous congruency at [80]. Similarly, there was only a Gratton effect at [80].

**Table 3:** **Error rates for each of the four 2 (previous congruency) x 2 (congruency) cells as a function of the proportion of congruent trials**.

**Condition**			**Proportion of congruent trials (%)**															
			**10**	**15**	**20**	**25**	**30**	**35**	**40**	**45**	**50**	**55**	**60**	**65**	**70**	**75**	**80**
Congruent-congruent			0.8	0.7	1.9	1.1	0.7	1.2	0.7	1.0	0.9	0.7	0.7	0.9	0.7	0.8	0.7
Congruent—incongruent			2.9	2.8	3.5	2.9	3.4	3.0	3.7	4.5	4.0	4.4	4.4	4.8	5.3	5.4	7.1
Incongruent-congruent			0.5	0.9	0.9	1.2	0.9	1.0	1.0	1.0	1.0	1.0	1.0	1.2	0.8	0.9	0.8
Incongruent-incongruent			2.3	2.0	2.4	2.5	2.4	2.4	2.6	2.8	2.3	3.0	3.3	3.3	2.8	3.9	2.9
**ANOVA EFFECTS**																	
Previous congruency	*F*_(1,14)_		1.12	0.21	4.57	0.20	0.70	2.54	0.58	3.17	1.94	2.25	0.96	1.39	3.39	1.60	**6.85**
	*p*		0.308	0.657	0.051	0.659	0.415	0.133	0.458	0.097	0.185	0.156	0.344	0.258	0.087	0.226	**0.020**
	mean ± std error		−0.4 ± 0.4	−0.3 ± 0.5	−1.1 ± 0.5	−0.2 ± 0.4	−0.4 ± 0.4	−0.4 ± 0.2	−0.4 ± 0.5	−0.8 ± 0.5	−0.8 ± 0.5	−0.5 ± 0.3	−0.4 ± 0.4	−0.6 ± 0.5	−1.2 ± 0.6	−0.7 ± 0.5	−**2.1 ± 0.8**
Congruency	*F*_(1, 14)_		**5.47**	**14.72**	**7.26**	**5.36**	**5.71**	2.98	**6.47**	**5.51**	**5.14**	**7.37**	**7.27**	**8.02**	**5.66**	**5.96**	**5.79**
	*p*		**0.035**	**0.002**	**0.017**	**0.036**	**0.032**	0.106	**0.023**	**0.034**	**0.040**	**0.017**	**0.017**	**0.013**	**0.032**	**0.029**	**0.030**
	mean ± std error		**1.9 ± 0.8**	**1.6 ± 0.4**	**1.6 ± 0.6**	**1.5 ± 0.6**	**2.1 ± 0.8**	1.6 ± 0.9	**2.3 ± 0.9**	**2.6 ± 1.1**	**2.2 ± 0.9**	**2.8 ± 1.0**	**3.0 ± 1.1**	**3.0 ± 1.0**	**3.3 ± 1.3**	**3.8 ± 1.5**	**4.3 ± 1.7**
Previous congruency × Congruency (Gratton Effect)	*F*_(1, 14)_		0.14	3.58	0.03	0.66	1.21	1.68	2.25	3.45	2.70	4.32	2.95	3.24	3.98	2.33	**7.88**
	*p*		0.711	0.079	0.869	0.429	0.291	0.216	0.156	0.084	0.122	0.056	0.108	0.094	0.066	0.149	**0.014**
	mean ± std error		0.3 ± 0.8	1.0 ± 0.5	0.1 ± 0.9	0.4 ± 0.5	1.1 ± 1.0	0.6 ± 0.4	1.5 ± 1.0	1.7 ± 0.9	1.8 ± 1.1	1.7 ± 0.8	1.5 ± 0.8	1.9 ± 1.0	2.6 ± 1.2	1.6 ± 1.0	**4.3 ± 1.5**

*The bottom portion of the table lists the parameter estimates obtained from the ANOVA testing for the presence of a Gratton effect. The bold values signify that *p* < *0.05**.

Despite the general absence of effects, for completeness the same repeated-measures regression analyses performed on the RT data were conducted here. The solid black circles in Figure 1B show the size of the Gratton effect as a function of the proportion of congruent trials. The results of this analysis reveal a slope of 0.014 ± 0.032 ms, *t*_(14)_ = 0.44, *p* > 0.50, and an intercept of −0.698 ± 0.802, *t*_(14)_ = −0.87, *p* < 0.40. This is shown as the solid black line in Figure 1B.

#### Excluding stimulus repetitions

Table 4 mirrors Table 2 with mean percent error rate in place of RTs. The same analysis contained in Table 2 is shown in the middle of Table 4. There was a significant main effect of congruency at each level of proportion congruency [10, 20, 25, 45, 55, 80]. There was a significant main effect of previous congruency only at [40, 65]. There were no significant Gratton effects.

**Table 4:** **Error rates for each of the four 2 (previous congruency) × 2 (congruency) cells as a function of the proportion of congruent trials after dividing them into whether they contain target->target, distractor->distractor, target->distractor, or distractor->target repetitions**.

**Condition**		**Repetition type**				**Proportion of congruent trials (%)**														
		**wW**	**cC**	**wC**	**cW**	**10**	**15**	**20**	**25**	**30**	**35**	**40**	**45**	**50**	**55**	**60**	**65**	**70**	**75**	**80**
**CONGRUENT–CONGRUENT**																				
^(1)^	BLUE_blue_→RED_red_					**1.5**	**0.5**	**2.1**	**1.3**	**0.7**	**1.2**	**0.6**	**1.2**	**1.0**	**0.6**	**0.7**	**0.8**	**0.8**	**0.8**	**0.8**
^(2)^	BLUE_blue_→BLUE_blue_	×	×	×	×	0.0	1.1	0.7	0.7	0.6	1.0	1.0	0.4	0.7	0.9	0.6	1.0	0.5	0.7	0.7
**CONGRUENT–INCONGRUENT**																				
^(3)^	BLUE_blue_→RED_green_					**2.9**	**3.5**	**4.5**	**3.7**	**3.9**	**4.2**	**4.8**	**5.6**	**4.3**	**6.0**	**5.5**	**6.0**	**6.5**	**6.6**	**8.8**
^(4)^	BLUE_blue_→BLUE_red_	×			×	4.2	2.2	2.3	1.6	2.2	1.9	2.4	3.6	3.4	1.9	2.9	3.0	3.6	3.8	3.3
^(5)^	BLUE_blue_→RED_blue_		×	×		1.8	2.1	3.0	2.4	3.5	2.2	2.7	2.9	4.1	3.7	3.8	4.0	4.2	4.6	6.8
**INCONGRUENT–CONGRUENT**																				
^(6)^	RED_blue_→GREEN_green_					**0.9**	**1.0**	**1.3**	**1.1**	**0.7**	**1.0**	**1.2**	**1.0**	**1.0**	**1.0**	**1.1**	**0.9**	**1.1**	**0.8**	**0.9**
^(7)^	RED_blue_→RED_red_	×		×		0.3	0.4	0.5	1.6	1.3	1.3	1.4	1.1	1.3	1.3	1.5	2.0	0.6	1.4	0.5
^(8)^	RED_blue_→BLUE_blue_		×		×	0.0	1.2	0.6	1.1	0.8	0.7	0.3	1.0	0.9	0.6	0.6	1.1	0.4	0.6	0.8
**INCONGRUENT–INCONGRUENT**																				
^(9)^	RED_blue_→GREEN_yellow_					**3.0**	**2.5**	**3.3**	**3.0**	**3.4**	**3.3**	**3.4**	**4.0**	**3.5**	**3.7**	**5.1**	**2.5**	**3.9**	**4.2**	**4.0**
^(10)^	RED_blue_→RED_green_	×				2.3	1.8	2.4	2.2	2.0	2.4	2.4	2.4	2.1	4.5	4.9	4.9	2.0	3.8	5.8
^(11)^	RED_blue_→GREEN_blue_		×			1.7	1.3	1.7	1.4	1.7	1.7	2.2	1.5	1.8	1.9	2.3	1.8	1.7	2.1	1.3
^(12)^	RED_blue_→RED_blue_	×	×			0.8	1.0	0.9	1.9	0.9	1.1	0.6	0.8	0.9	0.9	1.7	5.3	1.6	1.9	0.0
^(13)^	RED_blue_→GREEN_red_			×		3.2	3.8	3.3	4.4	3.8	3.8	4.3	4.7	3.1	4.0	5.4	5.3	3.6	8.1	2.5
^(14)^	RED_blue_→BLUE_green_				×	2.0	1.5	1.8	2.3	2.2	1.5	2.1	2.5	2.0	1.5	0.6	2.7	3.0	3.5	6.1
^(15)^	RED_blue_→BLUE_red_			×	×	1.9	1.5	2.7	2.0	1.4	2.1	2.1	1.8	1.8	3.1	1.3	0.4	3.3	0.0	0.0
**GRATTON ANOVA EFFECTS**																				
Previous congruency		*F*_(1, 14)_				4.44	1.31	4.22	0.07	4.24	3.83	**5.42**	2.93	2.63	0.01	0.11	**6.61**	0.99	0.04	0.01
		*p*				0.054	0.272	0.059	0.789	0.059	0.071	**0.035**	0.109	0.127	0.934	0.748	**0.022**	0.337	0.846	0.932
		mean ± std error				0.8 ± 0.4	0.5 ± 0.4	0.9 ± 0.4	0.1 ± 0.4	1.1 ± 0.5	0.7 ± 0.4	**1.3 ± 0.5**	1.2 ± 0.7	1.1 ± 0.6	0.1 ± 0.8	0.4 ± 1.1	**−3.0 ± 1.1**	1.0 ± 1.0	0.3 ± 1.4	−0.2 ± 2.7
Congruency		*F*_(1, 14)_				**8.05**	3.66	**8.03**	**6.26**	3.01	4.12	2.88	**8.7**	3.89	**5.43**	3.04	0.03	1.92	2.42	**6.74**
		*p*				**0.013**	0.076	**0.013**	**0.025**	0.105	0.062	0.112	**0.011**	0.069	**0.035**	0.103	0.867	0.188	0.142	**0.021**
		mean ± std error				**1.4 ± 0.5**	1.0 ± 0.5	**1.6 ± 0.5**	**1.0 ± 0.4**	1.5 ± 0.8	1.5 ± 0.7	1.5 ± 0.8	**2.0 ± 0.7**	1.4 ± 0.7	**2.7 ± 1.1**	3.0 ± 1.7	0.2 ± 1.2	1.3 ± 0.9	2.0 ± 1.2	**4.2 ± 1.6**
Previous congruency × Congruency (Gratton effect)		*F*_(1, 14)_				0.09	0.24	0.01	2.36	0.70	0.18	0.49	0.36	0.23	0.94	0.05	0.30	1.36	0.00	0.88
		*p*				0.772	0.635	0.944	0.146	0.416	0.676	0.497	0.557	0.640	0.348	0.832	0.594	0.263	0.972	0.365
		mean ± std error				0.2 ± 0.6	−0.3 ± 0.7	0.0 ± 0.6	−1.3 ± 0.8	−0.6 ± 0.7	−0.2 ± 0.6	0.6 ± 0.9	−0.9 ± 1.5	−0.5 ± 1.0	1.7 ± 1.7	0.5 ± 2.1	−1.0 ± 1.8	−1.9 ± 1.5	−0.1 ± 3.2	3.1 ± 3.2
**CONGRUENCY SWITCH ANOVA EFFECTS**																				
Switch		*F*_(1, 14)_				0.17	2.90	0.05	0.41	0.39	0.50	1.70	1.26	0.39	**10.37**	1.39	**5.15**	1.71	1.47	**14.12**
		*p*				0.684	0.110	0.826	0.534	0.540	0.491	0.214	0.280	0.540	**0.006**	0.257	**0.040**	0.212	0.246	**0.002**
		mean ± std error				−0.3 ± 0.8	0.8 ± 0.4	0.1 ± 0.6	0.3 ± 0.4	0.2 ± 0.4	0.4 ± 0.5	1.1 ± 0.8	0.7 ± 0.6	0.4 ± 0.7	**1.4 ± 0.4**	0.4 ± 0.4	**1.8 ± 0.8**	1.4 ± 1.1	1.2 ± 1.0	**2.5 ± 0.7**
Congruency		*F*_(1, 14)_				1.73	**7.79**	**6.11**	4.48	**5.44**	**5.50**	**7.10**	**5.84**	**4.97**	**5.45**	3.83	**4.71**	**5.58**	4.06	2.96
		*p*				0.210	**0.014**	**0.027**	0.053	**0.035**	**0.034**	**0.019**	**0.030**	**0.043**	**0.035**	0.071	**0.048**	**0.033**	0.064	0.107
		mean ± std error				1.8 ± 1.4	**2.2 ± 0.8**	**2.2 ± 0.9**	2.2 ± 1.0	**2.9 ± 1.3**	**2.6 ± 1.1**	**3.2 ± 1.2**	**3.7 ± 1.5**	**2.9 ± 1.3**	**4.1 ± 1.7**	4.4 ± 2.2	**3.4 ± 1.6**	**4.2 ± 1.8**	4.6 ± 2.3	5.6 ± 3.2
Switch × Congruency (Switch effect)		*F*_(1, 14)_				0.09	0.23	2.37	1.97	0.33	0.65	0.29	2.22	0.56	**5.51**	0.00	**6.28**	1.09	1.53	**10.32**
		*p*				0.766	0.640	0.146	0.182	0.574	0.435	0.601	0.158	0.467	**0.034**	0.945	**0.025**	0.315	0.237	**0.006**
		mean ± std error				−0.5 ± 1.5	−0.5 ± 1.0	−2.0 ± 1.3	−1.0 ± 0.7	−0.5 ± 0.9	−1.0 ± 1.3	−0.8 ± 1.5	−1.8 ± 1.2	−0.8 ± 1.1	−**1.9 ± 0.8**	−0.1 ± 0.9	−**3.5 ± 1.4**	−2.3 ± 2.2	−2.4 ± 1.9	−**4.7 ± 1.5**

*The middle portion of the table lists the parameter estimates obtained from the ANOVA testing for the presence of a Gratton effect, and the bottom portion of the table lists the parameter estimates obtained from the ANOVA testing whether the remaining Gratton effect results from the congruency switching hypothesis. Note: The absence of an interaction in the bottom portion is consistent with the switching hypothesis. The bold values signify that *p* < *0.05**.

Paired *t*-tests showed that, with the exception of [15, 25, 70] where the ps were marginal (0.095, 0.084, and 0.087 respectively), the size of the Gratton effects in Tables 3, 4 were statistically equivalent (*p*s > 0.15).

The solid gray circles in Figure 1B show the size of the Gratton effect as a function of the proportion of congruent trials. The same repeated measures regression analysis reported above yielded a slope of 0.038 ± 0.017, *t*_(14)_ = 2.26, *p* = 0.040, and an intercept of −0.250 ± 0.281, *t*_(14)_ = −0.89, *p* > 0.35. This significant slope is difficult to interpret given the fact that there is no significant Gratton effect in any of the proportion conditions. This is shown as the gray line in Figure 1B. Although the slope is larger and the intercept is smaller than in the previous analysis which included repetition effects, both are statistically equivalent to the previous parameter estimates, *p* = 0.614 for the slope and *p* = 0.627 for the intercept.

#### Can the switch hypothesis account for the remaining gratton effect?

Although there was no significant Gratton effect, a 2 (Switch) × 2 (Congruency) analysis was still conducted at each proportion level. Although these factors only interact at [55, 65, 80], it would be difficult to argue that Switch and Congruency are additive given the absence of a main effect of switch effect at all levels of proportion except the ones at which the factors interact, [55, 65, 80].

## Discussion

Experiment 1 reanalyzed a large-scale vocal Stroop study to assess the presence of a conflict adaptation component to the Gratton effect after ruling out three possible sources of binding confounds. There is no contingency bias in the 25% congruency condition. The analyses confirmed the presence of a Gratton effect in this condition, which could not be explained by the congruency switch hypothesis. Experiment 2 provides an independent replication of this result confirming that the Gratton effect remains after controlling for the three biases described by Schmidt and De Houwer (2011).

These analyses revealed two additional findings. First, the size of the Gratton effect in a vocal Stroop task is not affected by the contingencies between the color and the word as indicated by the statistically zero slopes in Figure 1A. Second, stimulus repetitions have little effect on the size of the Gratton effect in a vocal Stroop task. Although excluding stimulus repetition, in general, reduces the size of the effect, the real impact appears to be on the amount of variance in the size of the Gratton effect. That is, the average Gratton effect collapsed across proportions is 38 ± 5 ms with repetitions and 31 ± 8 ms without. These estimates are statistically equal (*p* > 0.30). However, the standard deviation of the size of the Gratton effect across proportions is 30 ± 3 ms with repetitions and 86 ± 10 ms without. These estimates are quite different, *t*_(14)_ = 6.04, *p* < 0.001. It is difficult to know whether the increase in variance occurs because of the fact that various stimulus repetition types account for 24.8, 50.5, 49.8, and 83.0% of trials in the CC, CI, IC, and II conditions respectively, or due an unknown psychological construct.

### Potential issues

The astute reader will have identified one potentially important problem with these re-analyses: the proportion of congruent trials—the color-word contingency—was manipulated within-subjects. So, even though a given block of 100 trials in the middle of the session may have a chance-level contingency (i.e., the 25% blocks) between the word and the color, that block was preceded by many blocks of trials in which the contingency between the color and the word was greater that chance.

As noted in the methods, each subject received the same random block order. Coincidently, the first block was the 25% congruent condition. Thus, to avoid any possible longer-term association confounds, analyses looking only at this first block of 100 trials^1^ were conducted both including and excluding repetitions. A Gratton effect measuring 42 ± 14 ms was observed with repetitions, *F*_(1, 13)_ = 8.76, *p* = 0.011, and measuring 52 ± 18 ms was observed without repetitions, *F*_(1, 13)_ = 8.44, *p* = 0.012. In addition, the remaining Gratton effect cannot be explained by the congruency switch hypothesis. Specifically, response times, excluding repetitions, yields a 70 ± 28 ms congruency switch x congruency interaction *F*_(1, 13)_ = 6.83, *p* = 0.020 comprised of a Stroop effect of 99 ± 20 ms, *t*_(13)_ = 5.27, *p* < 0.001, following a congruency repetition and 29 ± 20 ms, *t*_(13)_ = 1.53, *p* = 0.153, following a congruency switch.

## Experiment 2: a gratton analysis of the 25% congruency condition

Excluding feature repetitions in Experiment 1 raised an important issue; the absence of a reliable reduction in the Gratton effect when feature repetitions are excluded is inconsistent with the results of previous studies on this issue. We suspect this may have occurred because (1) we had a relatively small sample size for addressing this issue and (2) because proportion was manipulated within-subjects, it may be that subjects actually formed non-zero contingencies between the color and word pairs. To address these concerns, we conducted a new experiment with the goal of replicating these results with a larger sample size and using only the 25% congruency condition. The results from Experiment 2 indicated that there is still a strong Gratton effect after stimulus repetition trials are removed that cannot be explained by a congruency switch, suggesting that color-word contingency plays no role in the Gratton effect.

### Methods

For full methodological details, see Blais et al. (2010). Briefly, thirty subjects were asked to perform 820 trials in a vocal Stroop task. The first 20 trials were considered practice and were used to calibrate the microphone and not included in any of the reported analyses. The remaining 800 trials were divided into four blocks of 200 trials with a self-paced break between them. Importantly, the trials were 25% congruent; 200 trials were congruent and 600 were incongruent, thus any of the four words were equally likely to appear in any of the four colors thereby eliminating all word-color association biases.

## Results

### Analyses of RTs

#### Including stimulus repetitions

The same 2 (previous congruency) by 2 (current congruency) ANOVA reported in Experiment 1 was conducted on these data. The results are shown in Table 5. There was a significant main effect of congruency, with congruent trials (761 ms) responded to faster than incongruent trials (833 ms), and a significant main effect of previous congruency, with trials on which the previous trial was congruent (783 ms) being responded to faster than trials on which the previous trial was incongruent (811 ms). Critically, these factors interacted to produce a Gratton effect, *F*_(1, 29)_ = 15.9, *p* < 0.001: the Stroop effect was larger following congruent trials (92 ± 11 ms) than following incongruent trials (54 ± 8 ms).

**Table 5:** **Response times and error rates for each of the four 2 (previous congruency) × 2 (congruency) cells in Experiment 2**.

**Condition**		**Measure**	
		**RT**	**% errors**
Congruent-congruent		740	0.5
Congruent—incongruent		831	2.9
Incongruent-congruent		784	0.4
Incongruent-incongruent		838	2.0
**ANOVA EFFECTS**			
Previous congruency	*F*_(1, 29)_	**20.5**	**8.7**
	*P*	**<0.001**	**0.006**
	mean ± std error	**26 ± 6**	−**0.6 ± 0.2**
Congruency	*F*_(1, 29)_	**79.0**	**49.1**
	*P*	**<0.001**	**<0.001**
	mean ± std error	**73 ± 8**	**1.9 ± 0.3**
Previous congruency × Congruency (Gratton effect)	*F*_(1, 29)_	**14.0**	**5.0**
	*p*	**<0.001**	**0.033**
	mean ± std error	**37 ± 10**	**0.8 ± 0.4**

*The bottom portion of the table lists the parameter estimates obtained from the ANOVA testing for the presence of a Gratton effect. The bold values signify that p < 0.05*.

#### Excluding stimulus repetitions

The same 2 (previous congruency) by 2 (current congruency) ANOVA reported in Experiment 1, but excluding stimulus repetitions, was conducted on these data. The results are shown in Table 6. There was a significant main effect of congruency, with congruent trials (795 ms) responded to faster than incongruent trials (867 ms), *F*_(1, 29)_ = 57.6, *p* < 0.001 and a significant main effect of previous congruency, with trials on which the previous trial was congruent (817 ms) being responded to faster than trials on which the previous trial was incongruent (845 ms), *F*_(1, 29)_ = 14.1, *p* < 0.001. Critically, these factors interacted to produce a Gratton effect, *F*_(1, 29)_ = 10.1, *p* < 0.005: the Stroop effect was larger following congruent trials (89 ± 11 ms) than following incongruent trials (56 ± 10 ms).

**Table 6:** **Response times and error rates for each of the four 2 (previous congruency) × 2 (congruency) cells in Experiment 2 after dividing them into whether they contain target->target, distractor->distractor, target->distractor, or distractor->target repetitions**.

**Condition**		**Repetition type**				**Measure**	
		**wW**	**cC**	**wC**	**cW**	**RT**	**% errors**
**CONGRUENT–CONGRUENT**							
^(1)^	BLUE_blue_→RED_red_					**773**	**0.6**
^(2)^	BLUE_blue_→BLUE_blue_	×	×	×	×	644	0.4
**CONGRUENT–INCONGRUENT**							
^(3)^	BLUE_blue_→RED_green_					**863**	**3.1**
^(4)^	BLUE_blue_→BLUE_red_	×			×	823	2.1
^(5)^	BLUE_blue_→RED_blue_		×	×		777	3.0
**INCONGRUENT–CONGRUENT**							
^(6)^	RED_blue_→GREEN_green_					**817**	**0.3**
^(7)^	RED_blue_→RED_red_	×		×		800	0.4
^(8)^	RED_blue_→BLUE_blue_		×		×	697	0.2
**INCONGRUENT–INCONGRUENT**							
^(9)^	RED_blue_→GREEN_yellow_					**878**	**2.1**
^(10)^	RED_blue_→RED_green_	×				859	1.9
^(11)^	RED_blue_→GREEN_blue_		×			764	1.5
^(12)^	RED_blue_→RED_blue_	×	×			698	0.6
^(13)^	RED_blue_→GREEN_red_			×		894	2.4
^(14)^	RED_blue_→BLUE_green_				×	846	1.3
^(15)^	RED_blue_→BLUE_red_			×	×	873	2.8
**GRATTON ANOVA EFFECTS**							
Previous Congruency		*F*_(1, 29)_				**16.2**	**5.2**
		*p*				**<0.001**	**0.030**
		mean ± std error				**29 ± 7**	**−0.6 ± 0.3**
Congruency		*F*_(1, 29)_				**57.9**	**31.9**
		*p*				**<0.001**	**<0.001**
		mean ± std error				**76 ± 10**	**2.1 ± 0.4**
Previous congruency × Congruency (Gratton effect)		*F*_(1, 29)_				**4.8**	1.7
		*p*				**0.036**	0.204
		mean ± std error				**28 ± 13**	0.7 ± 0.6
**CONGRUENCY SWITCH ANOVA EFFECTS**							
Switch		*F*_(1, 29)_				**4.8**	1.7
		*p*				**0.036**	0.204
		mean ± std error				**14 ± 6**	−0.4 ± 0.3
Congruency		*F*_(1, 29)_				**57.9**	**31.9**
		*p*				**<0.001**	**<0.001**
		mean ± std error				**76 ± 10**	**2.1 ± 0.4**
Switch × Congruency (Switch effect)		*F*_(1, 29)_				**14.5**	**5.2**
		*p*				**<0.001**	**0.030**
		mean ± std error				**58 ± 14**	**1.2 ± 0.6**

*The middle portion of the table lists the parameter estimates obtained from the ANOVA testing for the presence of a Gratton effect, and the bottom portion of the table lists the parameter estimates obtained from the ANOVA testing whether the remaining Gratton effect results from the congruency switching hypothesis. Note: The absence of an interaction in the bottom portion is consistent with the switching hypothesis. The bold values signify that p < 0.05*.

#### Can the switch hypothesis account for the remaining gratton effect?

To assess whether the remaining Gratton effect is the result of congruency switching (i.e., Schmidt and De Houwer, 2011), a 2 (Switch) × 2 (Congruency) analysis was conducted on data after excluding stimulus repetitions. Again, an in-depth description of the logic of this analysis is provided by Schmidt and De Houwer (2011). In short, additive effects of congruency switch (whether the congruency on the previous trial is the same, or different, on the current trials) and congruency are consistent with an interpretation in which the Gratton effect results from a reconfiguration switch cost. An interaction of these factors cannot rule out a conflict adaption account of the Gratton effect. There was a main effect of congruency, with congruent trials (795 ms) responded to faster than incongruent trials (867 ms), *F*_(1, 29)_ = 57.6, *p* < 0.001. There was a switch cost, congruency switch trials (839 ms) were responded to slower than congruency repetition trials (823 ms), *F*_(1, 29)_ = 10.1, *p* < 0.005. Critically, these factors interact, rather than add, *F*_(1, 29)_ = 14.1, *p* < 0.001, the Stroop effect is larger on congruency repetition trials (101 ± 12 ms) vs. congruency switch trials (45 ± 12 ms), thereby ruling out the switch hypothesis as an explanation for the remaining Gratton effect.

### Analyses of errors

The overall rate rates was only 1.4%, but for the sake of completeness, the entire set of analyses performed on RTs was also done on error rates.

#### Including stimulus repetitions

The same 2 (previous congruency) by 2 (current congruency) ANOVA reported in Experiment 1 was conducted on these data. There was a significant main effect of congruency, with congruent trials (0.4%) responded to more accurately than incongruent trials (2.3%), and a significant main effect of previous congruency, with trials on which the previous trial was congruent (1.2%) being responded to more accurately than trials on which the previous trial was incongruent (1.7%), *F*_(1, 29)_ = 7.5, *p* < 0.05. Critically, these factors interacted to produce a Gratton effect; the Stroop effect was larger following congruent trials (2.4 ± 0.4%) than following incongruent trials (1.5 ± 0.2%).

#### Excluding stimulus repetitions

The same 2 (previous congruency) by 2 (current congruency) ANOVA reported in Experiment 1, but excluding stimulus repetitions, was conducted on these data. There was a significant main effect of congruency, with congruent trials (0.5%) responded to more accurately than incongruent trials (2.6%), and a significant main effect of previous congruency, with trials on which the previous trial was congruent (1.8%) being responded to less accurately than trials on which the previous trial was incongruent (1.2%). These factors failed to interact to produce a Gratton effect; the Stroop effect was statistically equivalent following congruent trials (2.5 ± 0.6%) than following incongruent trials (1.8 ± 0.3%).

#### Can the switch hypothesis account for the remaining gratton effect?

To assess whether the remaining Gratton effect is the result of congruency switching (i.e., Schmidt and De Houwer, 2011), a 2 (Switch) × 2 (Congruency) analysis was conducted on data after excluding stimulus repetitions. Again, an in-depth description of the logic of this analysis is provided by Schmidt and De Houwer (2011). In short, additive effects of congruency switch (whether the congruency on the previous trial is the same, or different, on the current trials) and congruency are consistent with an interpretation in which the Gratton effect results from a reconfiguration switch cost. An interaction of these factors cannot rule out a conflict adaption account of the Gratton effect. There was a main effect of congruency, with congruent trials (0.5%) responded to faster than incongruent trials (2.6%). There was no switch cost; congruency switch trials (1.7%) were responded equivalently to congruency repetition trials (1.3%). Critically, these factors interact, rather than add; the Stroop effect is larger on congruency repetition trials (2.8 ± 0.6%) vs. congruency switch trials (1.5 ± 0.3%), thereby ruling out the switch hypothesis as an explanation for the remaining Gratton effect.

## Discrepancies with Schmidt and De Houwer

What seems clear from the current set of experiments using a vocal Stroop task is that at least part of the Gratton effect results from conflict adaptation. The vocal Stroop task has been called the gold standard of attention measures (e.g., MacLeod, 1992). Anyone who has done a standard vocal Stroop task knows how difficult it is to not blurt out the word. That is, individuals must suppress an extremely strong, obligatory urge to read the word. That is, it seems fairly obvious that the desire to read aloud the word is the major source of conflict. Thus, conflict adaptation is more likely in this task because conflict is more pervasive, and hence more disturbing, for participants (e.g., Desender et al., 2014).

In the manual Stroop task and the flanker task, the source of conflict is less clear. That is, it seems that there are at least two major sources of conflict. The first source is the distractor item. The presence of Stroop and flanker effects is consistent with this interpretation. But, there must be at least one other source of conflict given that many errors are random in the sense that subjects are not responding to the distractor feature, but to a non-presented feature (i.e., the participant said “red” to BLUE_green_). For example, in a four-choice flanker task, you can make an error by hitting any one of the three non-target keys. If errors are completely random, one would expect a 33.3% chance of hitting any of them. As it turns out, subjects tend to make significantly more “distractor errors”; 46.6% compared to 26.7% (e.g., see Maier et al., 2011). Although significant, subjects still make a majority of these random errors, perhaps because they are responding too quickly. In the vocal Stroop task, this type of error is rare. In fact, Experiment 1, containing over 270,000 trials, there were less than a dozen errors of this nature where “blue” was said to YELLOW_red_. The high random error rate in the manual tasks is consistent with the idea that subjects have a much weaker internal representation of which key is associated with which response. This continual need for control to maintain the response sets likely creates a second source of conflict.

Errors in the vocal Stroop task are rare, but errors are among the most subjectively salient need-for-control cues. Behaviorally, they yield a post-error slowing effect on subsequent trials (Rabbitt, 1966; Laming, 1979; Unsworth et al., 2012). This slowdown has been correlated with activity in anterior cingulate cortex (e.g., Yeung et al., 2003). In fact, this error-related negativity is often strong enough to be observed on single trials in unprocessed event related potentials (ERPs) and fMRI BOLD signals. However, Schmidt and De Houwer fail to exclude such trials from their analysis. Perhaps more important than post-error slowing is the observation that errors are often followed by a reduction of conflict effect producing a Gratton-like effect. For instance, Maier et al. (2011) have shown that this effect occurs only after incongruent flanker errors.

Therefore, there are two additional sources of conflict that might better be characterized as sustained control in the manual data. First, subjects must maintain, relatively unpracticed, button mappings. This source of conflict is unlikely to vary trial-to-trial. Second, these weak button maps lead to a large number of errors. Indeed, the subjects in Experiment 1 from Schmidt and De Houwer (2011) made approximately 13% errors overall; our subjects were <2%. Critically, trials following errors are slowed. This will obviously vary from trial-to-trial, but Schmidt and De Houwer failed to remove this source of conflict^2^. In theory, this should lead to the observation of a Gratton effect, but due to the unusually high number of errors overall, it is unclear if hypothesis this will hold.

## Conclusion

Starting with the original Mayr et al. (2003) paper, there has been a heated debate as to whether conflict adaptation plays a role in the Gratton effect over and above feature repetition binding biases. The answer to this question appears to be yes (e.g., Ullsperger et al., 2005). Schmidt and De Houwer (2011) identified two additional binding confounds: inherent contingencies between the relevant and irrelevant dimensions, and congruency switching effects. Using manual versions of the Stroop and flanker tasks, they make the strong claim that conflict adaptation is not necessary to explain the Gratton effect. Yet in a vocal Stroop task, we show that nearly all of the Gratton effect is the result of conflict adaption. The more important question, however, is whether conflict adaptation occurs “in real life”; not in any particular lab task. Further research is necessary to allow us to identify the role of conflict adaptation in more naturalistic situations.

### Conflict of interest statement

The authors declare that the research was conducted in the absence of any commercial or financial relationships that could be construed as a potential conflict of interest.

## Footnotes

^1^One subject was dropped from the analysis because they had no observations in the congruent-congruent condition after excluding repetition trials.

^2^Excluding trials following errors has no qualitative impact on our findings.
